# Prediction-based sensory attenuation is related to prediction-based motor attenuation

**DOI:** 10.1162/IMAG.a.1045

**Published:** 2025-12-05

**Authors:** Dominic M.D. Tran, Nicolas A. McNair, Alexis E. Whitton, Thomas J. Whitford, Evan J. Livesey

**Affiliations:** The University of Sydney, Camperdown, NSW, Australia; Black Dog Institute, Randwick, NSW, Australia; UNSW Sydney, Kensington, NSW, Australia

**Keywords:** predictive coding, prediction, associative learning, sensory attenuation, motor system, transcranial magnetic stimulation, TMS-EEG

## Abstract

When sensory inputs can be predicted by an organism’s own actions or external environmental cues, neural activity is often attenuated compared with sensory inputs that are unpredictable. We have recently demonstrated that attenuation to predictable inputs is also observed when stimulating the motor system with transcranial magnetic stimulation (TMS). Akin to sensory attenuation, the motor system is less responsive to predictable TMS compared with unpredicted TMS. However, it remains unclear whether these motor prediction signals are related to, or even dependent on, sensory prediction. Using a two-coil single-pulse TMS setup to target distinct brain regions, we arranged different warning cues to predict different regions of stimulation and measured motor attenuation using motor-evoked potentials. We found that expecting TMS over the motor cortex produced stronger attenuation than expecting TMS over a non-motor region, confirming that the attenuation observed is directly linked to activation of the motor system and not due to the sensory by-products of TMS. Using combined TMS-EEG, we measured motor attenuation with motor-evoked potentials, and simultaneously measured sensory attenuation to the sound of TMS (a coil “click”) with auditory-evoked potentials. We found that both motor and auditory potentials were attenuated to predictable TMS compared with unpredictable TMS. Critically, the magnitude of auditory attenuation predicted the magnitude of motor attenuation. Our results reveal a close correspondence between prediction processing in the sensory and motor systems. The findings provide evidence consistent with predictive coding being governed by domain-general properties across distinct neural systems, suggesting there could be common mechanisms responsible for different forms of predictive learning.

## 1 Introduction

Prediction-based attenuation of the brain’s response to sensory inputs is widely observed across different modalities and organisms. When sensory inputs such as auditory (Mifsud & Whiteford, 2017; Sowman et al., 2012), visual (Kok et al., 2012; Tang et al., 2023), or tactile (Blakemore, Rees, & Frith, 1998) information can be predicted by an organism’s own actions or by external warning cues, neural activity is attenuated compared with sensory inputs that are unpredictable. For example, when moving our hand to touch another part of our body, we can predict haptic information from an efference copy of the signal sent to move our hand (e.g., Wolpert et al., 1995). This efference copy allows the tactile sensation to be anticipated in advance of the hand touching the body (i.e., corollary discharge; Sperry, 1950), and results in a less salient sensation being experienced compared with an external source touching the body. This form of sensory attenuation to predictable information is thought to be the reason we are unable to tickle ourselves (Blakemore, Wolpert, & Frith, 1998).

Sensory attenuation is adaptive from an evolutionary standpoint. Attenuation of predictable or self-generated sensory information allows us to prioritise unexpected and novel sensory information. Such functions are likely to be fundamental and ubiquitous to the central nervous system given demonstrations of sensory attenuation in many animals, including insects, fish, birds, and mammals (Crapse & Sommer, 2008). Meanwhile dysfunctions in sensory attenuation in humans have also been linked with atypical thoughts and cognitions, with impairments in sensory attenuation, for example, linked with schizotypy traits (Oestreich et al., 2015) and schizophrenia symptoms (Ford et al., 2007).

Modelled in the laboratory, sensory attenuation can be measured using an auditory response task in which a sound is played when the participant presses a key or the sound is played without warning (e.g., Ford et al., 2007). Sounds are accompanied by a characteristic auditory-evoked potential when neural activity is measured using EEG. The amplitude of the auditory-evoked N1 component is smaller when sounds are delivered predictably and is larger when sounds are delivered unpredictably (e.g., Mifsud et al., 2016; Oestreich et al., 2015). Critically, the loudness of these sounds is matched, but it is their predictability that modulates the magnitude of the N1 component. This attenuation is also sometimes reported in the P2 component (e.g., Harrison et al., 2021). These results suggest that the brain’s neural response to predictable auditory signals is weaker than unpredictable auditory signals.

We recently demonstrated that prediction-based attenuation is also present in the motor system (Tran et al., 2021; Tran & Livesey, 2021; see also Sriutaisuk & Franz, 2025; Takei et al., 2005). Using transcranial magnetic stimulation (TMS), we stimulated the left motor cortex and measured motor-evoked potentials (MEP) recorded in the contralateral (right) hand. Across three experiments, we showed that direct stimulation of the motor cortex produced weaker MEPs, or motor attenuation, when the stimulation was predictable compared with when the stimulation was unpredictable (Tran et al., 2021). Notably, in Experiment 2, TMS was delivered under three different conditions (1) triggered by the participant (self-generated), 2) triggered shortly after a visual stimulus (warning cue), or (3) triggered with no warning (unpredictable baseline). We showed that MEPs were attenuated in both the predictable conditions (self-generated and warning cue) compared with the unpredictable condition, while the self-generated condition produced stronger attenuation than the warning cue condition. We also found that conditions predicting stimulation 100% of the time resulted in stronger attenuation than conditions predicting stimulation 25% of the time; these conditions were matched for the actions made by the participant and differed only in the stimulation probability. Notably, the attenuation was observed to a novel event (i.e. TMS) with which most participants had no experience; TMS bypassed sensory channels to directly activate the motor cortex; and the effect did not rely on participants making any motor responses (see Tran et al., 2021, Experiment 3; Tran & Livesey, 2021). This suggests that the brain’s predictive mechanisms are ubiquitous and sufficiently flexible to adapt to completely novel stimuli. If the function of a neural network is to process incoming but noisy information and return a meaningful output, then attenuating less informative parts of a signal is generally beneficial, whether it is sensory, motor, or any other information.

Other neural systems also possess functional characteristics that mirror prediction-based attenuation. For instance, the seminal findings by Schultz et al. (1997) can be interpreted as showing that dopamine neurons recorded from the ventral tegmental area of monkeys fired less to predicted delivery of juice compared with unpredicted delivery of juice. Similar effects have been observed in the ventral pallidum neurons of rats using a cued reward task with sucrose (Ottenheimer et al., 2020). These studies provide evidence that neurons both within and outside of the midbrain show a form of prediction-based attenuation (Ottenheimer et al., 2020; Schultz et al., 1997), firing more to unexpected events and less to expected events. Although the methods and measurements are quite different to those reported in the sensory and motor attenuation literature discussed so far, the pattern of data is remarkably similar.

Taking together the evidence of prediction-based attenuation in sensory, motor, and reward systems, it suggests there may be a common mechanism that operates throughout the brain to down-weight predictable information and/or upweight unpredictable information. Such a mechanism could be a form of predictive coding that constantly generates mental models of the world from bottom-up information (e.g., Friston, 2005). Unexpected signals that are inconsistent with top-down model predictions—that is, prediction errors—are important for signalling that the current model does not accurately reflect the environment and requires updating. Attenuating predictable information may be one means for prioritising prediction error signals. Critically, most predictive coding theories are purely grounded in sensory processing, most often visual perception, but our recent results suggest that these predictive coding mechanisms may be domain general and operate using sensory, motor, or reward information in the same way. Few studies have tested this hypothesis, instead studying predictive coding phenomena by measuring output from only one domain. For example, although sensory attenuation effects can be studied with a motor component (i.e., press condition), the effect is typically measured within the sensory domain (e.g., using N1 auditory-evoked potentials). Similarly, previous studies that have examined motor-related attenuation using proprioceptive or somatosensory feedback have measured output from the sensory domain in the form of touch or tactile information, rather than directly measuring motor activity (e.g., Shergill et al., 2003).

In the current series of experiments, we tested the hypothesis that prediction-based neural attenuation is a domain-general property of the brain by comparing prediction-based attenuation of motor and auditory processes. Our hypothesis is that predictive mechanisms operate in a similar way across brain regions such that, regardless of the nature of the information that the region processes, predicted inputs are down-weighted and the neural response to those inputs is thus attenuated. While the nature of the prediction may be domain specific (e.g. conveying information about sensations, rewards, or motor commands), the mechanism itself is functionally similar. If this is the case, then we further hypothesise that inter-individual differences in this predictive mechanism might mean that an individual exhibits relatively strong or relatively weak attenuation across multiple domains. In order to test that this hypothesis is plausible, we first need to demonstrate that our motor attenuation effect is driven by predictions about stimulation of the motor system specifically, but that individual differences in attenuation nonetheless span different domains.

The first experiment tested whether previous demonstrations of motor attenuation were driven by prediction of the accompanying “click” sound and tactile scalp sensation produced with TMS. To rule out this alternative explanation, we used a two-coil single-pulse setup: one coil stimulated M1 while producing the typical sensory stimulation, and a second coil was positioned over the right parietal region (approximately over P4 EEG electrode site) to mimic the sensory by-products but did not stimulate M1. The control location was selected because stimulation at this location did not stimulate the motor cortex, did not interfere with visual processing, and could be positioned without touching the motor cortex coil. For the participant, the sensory by-products of TMS delivered over M1 have no a priori connection with motor control, that is, they have no reason to associate sounds or tapping sensations located over that region of the scalp with motor responses or motor preparation. Thus, we hypothesised that any effect of predicting sensory qualities of the TMS would not be based on these sensations being localised near M1. The aim of this experiment was to determine whether expecting stimulation over a control site produced the same degree of attenuation as expecting stimulation over the motor cortex. A key feature of the design is that both coils produce salient sensory by-products during stimulation (an auditory coil “click” and tactile vibration on the scalp), but only one coil reliably stimulated the motor cortex. Hence, if our previous attenuation findings (Tran et al., 2021) are the result of predicting the sensory by-products of TMS, anticipation of the control coil stimulation should also contribute to MEP attenuation. However, if our previous findings reflect attenuation of the motor system that is generated by predicting stimulation of the motor cortex, anticipation of the motor coil stimulation should produce stronger MEP attenuation. The results test whether MEP attenuation reflects down-weighting of neural activity specific to the prediction of motor cortex stimulation, or whether MEP attenuation reflects a reaction to the sensory expectation of brain stimulation.

The second experiment tested whether attenuation in the motor domain was related to attenuation in the sensory domain by simultaneously measuring prediction-based attenuation from the motor system using TMS, and from the auditory system using EEG. To investigate this, we used a combined TMS-EEG setup to measure MEPs and auditory-evoked potentials (AEPs; a type of event-related potential or ERP). Critically, the TMS stimulation was used to measure attenuation in the motor domain, but the sound generated by the TMS pulse was used as the auditory stimulus to measure attenuation in the sensory domain. The setup, therefore, allows the simultaneous measurement of motor attenuation to motor cortex stimulation using MEPs and sensory attenuation to the auditory click from the same TMS pulse using AEP. The task was similar to the standard auditory response task used to measure sensory attenuation, but TMS pulses replaced the sounds. Mimicking this design, TMS could be triggered by the participant, followed by a warning cue, or triggered spontaneously without warning. If predictive coding is governed by domain general mechanisms, and these mechanisms vary in strength across individuals, we should see a close correspondence across individuals in the degree to which they show motor and sensory attenuation. That is, we would expect that individuals who show stronger sensory attenuation will also show stronger motor attenuation.

## 2 Methods

### 2.1 Design

Experiment 1 used a two-coil setup to examine the effect of stimulation predictability on MEP attenuation across three cue conditions. One cue reliably predicted motor cortex stimulation with 100% probability (M1 cue); one cue predicted stimulation over the control location with 80% probability (P4 cue) and stimulation of the motor cortex on the remaining 20% for measurement trials (note that TMS over a non-motor region cannot produce MEPs, so to measure motor attenuation, the P4 cue needs to be followed by M1 stimulation some of the time); and one cue predicted no stimulation with 80% probability (control cue) and stimulation of the motor cortex on the remaining 20% as a probability control for the P4 cue (see Table 1). The P4 and the control cues both stimulate the motor cortex 20% of the time, but the P4 cue is always accompanied by stimulation (the rest of the stimulation was over the parietal lobe). If MEP attenuation is the result of expecting motor cortex stimulation, the P4 and control cues should produce equivalent attenuation since both cues stimulate the motor cortex 20% of the time. However, if MEP attenuation is driven by expectation of the by-products of TMS, the M1 and P4 cues should produce equivalent attenuation since both cues trigger a TMS pulse on every trial. There were a total of 240 M1 TMS trials (150 M1 cue, 30 P4, 30 control cue, 30 unpredictable baseline) and 120 P4 TMS trials.

**Table 1.** Experiment 1 design showing the stimulation probability by cue condition.

	M1 stimulation probability	P4 stimulation probability	Sensory probability
M1 cue	100%	0%	100%
P4 cue	20%	80%	100%
Control cue	20%	0%	20%

*Note.* The combined stimulation probabilities result in a corresponding sensory probability. The P4 and control cues are matched for M1 stimulation probability, while the P4 and M1 cues are matched for sensory probability.

Experiment 2 used a combined TMS-EEG setup to examine the relationship between motor and sensory attenuation across three predictability conditions. One condition asked participants to make a key press following the presentation of a visual cue, and doing so triggered the TMS (self-generated); one condition triggered the TMS shortly after the presentation of a different visual cue (warning cue); and one condition triggered the TMS spontaneously without warning (unpredictable baseline). There were a total of 324 EEG trials across three cue conditions. Half of the EEG trials were accompanied by TMS stimulation and half of the EEG trials involved the associated cues and actions of each condition but without any stimulation. Taking a difference wave between the TMS and no-TMS EEG trials removes any neural activity resulting from the cue presentation or action generation, leaving only the neural activity resulting from the TMS pulse.

### 2.2 Participants

All participants completed a TMS safety questionnaire based on Rossi et al. (2009) and provided informed consent before starting the experiment. All protocols were approved by the Human Research Ethics Committee of The University of Sydney.

Experiment 1 was conducted on 46 participants with 1 participant withdrawing due to discomfort from the stimulation. Based on an estimated medium effect size of d = 0.5 (Tran et al., 2021; Experiment 2, probability effect), 34 participants are needed to achieve 80% power for detecting a within-participant effect of probability (difference in attenuation between the high predictability M1 cue and the low predictability control cue). We aimed to collect 45 participants to have approximately 40 participants remaining after exclusion since the effect of coil (difference in attenuation between the M1 cue and the P4 cue) could be smaller than the effect of probability. After excluding 4 participants who failed the attention check (see Procedure), there were 41 participants remaining for the analysis.

Experiment 2 was conducted on 40 participants. Based on an estimated large effect size of d = 0.8 (Tran et al., 2021; Experiment 2, agency effect), 15 participants are needed to achieve 80% for detecting a within-participant effect of prediction cue type. Based on a converted large effect size of ρ = 0.5, 26 participants are needed to achieve 80% for detecting a correlation between attenuation types. One participant had EEG recorded at an incorrect sampling rate and their data were not analysed; 39 participants remained for analysis of either the MEP or ERP data. Four participants were excluded from the MEP analysis because MEPs could not be reliably elicited after increasing the stimulator up to a comfortable intensity for the participant; due to the combined TMS-EEG setup, the TMS coil was further away from the scalp compared with a standard TMS experiment and, therefore, the average intensity was higher than a typical TMS experiment. Nine participants were excluded from the ERP analysis (see EEG Pre-processing and Analysis section). After exclusions, 35 participants remained for the MEP analysis, 31 participants remained for the ERP analysis, and 28 participants had both MEP and ERP data.

### 2.3 Apparatus and stimuli

Experiments were run on a Windows 7 PC using PsychoPy3 to control stimulus presentation. Stimuli were presented on a 24-inch monitor (1920 × 1080 resolution, 60 Hz refresh rate) at a viewing distance of ~57 cm. The stimuli presented on screen subtended approximately 2° of visual angle.

### 2.4 EMG

Three electrodes were attached to the right hand for electromyography (EMG) recording. In preparation for recording, the skin was exfoliated with a small scouring pad and wiped with 70% v/v isopropyl alcohol. Two 10-mm diameter Ag/AgCl electrodes were placed in a belly tendon arrangement over the first dorsal interosseus (FDI) muscle to measure MEPs. A ground electrode was placed over the ulnar styloid process of the wrist. EMG activity was recorded from 100 ms pre-stimulation to 400 ms post-stimulation. This signal was digitally converted (sampling rate: 4 kHz, bandpass filter: 0.5 Hz to 2 kHz, mains filter: 50 Hz, and anti-aliasing) and stored offline for analysis using LabChart software (Version 8, ADInstruments).

### 2.5 TMS

TMS was administered using a MagStim 200^2^ stimulator and a 70 mm D70^2^ figure-8 coil. Participants wore an elastic cap marked with the 10/20 EEG electrode positions to help locate the hand region of the motor cortex. The coil was held tangentially to the scalp with the coil oriented 45º from the midline. The motor cortex “hotspot” was located by starting from a position 5 cm lateral and 1 cm anterior to Cz. The coil was then moved around until the maximal MEP could be elicited in the FDI. Once the hotspot was determined and marked, the participants were asked to place their head on a chin and forehead rest for the coil to be locked in position with an adjustable mechanical arm (Manfrotto). Resting motor threshold (rMT) was defined as the lowest stimulation intensity that produced an MEP of at least 50 µV in 5 out of 10 consecutive trials (Rossini et al., 2015).

During the experiments, the stimulation intensity was set to 120% of rMT unless the participant requested a lower intensity due to discomfort (usually 110% of rMT). Participants were asked to keep their head still during thresholding and while the experiment was in session, but they could move any other time.

For the two-coil single-pulse setup (Experiment 1), a second 70 mm figure-8 coil was positioned approximately over the location of the P4 electrode under the 10/20 positioning standards. This coil was connected to a second MagStim 200^2^ stimulator and the stimulation intensity was set to the same value as the first stimulator. The mean stimulation intensity from all participants who started the experiment was M = 52.78% of the maximum stimulator output, SD = 11.37 (n = 46).

For the combined TMS-EEG set-up (Experiment 2), the TMS coil was covered with a 10 mm layer of pliable foam to minimise vibration of the coil on the EEG electrodes. The mean stimulation intensity from all participants who started the experiment was M = 75.15, SD = 12.29 (n = 40). Note that the mean stimulation intensity is higher than that in Experiment 1 due to the extra distance separating the coil and the scalp due to the addition of the foam and EEG electrodes.

### 2.6 EEG

In Experiment 2, EEG data were sampled continuously at 10,000 Hz from 64 Ag/AgCl active electrodes (actiCHamp, Brain Products, Gmbh, Gilching, Germany). The electrodes were situated in an EasyCap (GmbH, Woerthsee-Etterschlag, Germany) that positioned them approximately in standard 10/20 locations. Impedances were kept below 10kΩ. Data were acquired using electrode FCz as the online reference and then later re-referenced offline to the average of all 64 scalp electrodes.

### 2.7 Procedure

#### 2.7.1 Experiment 1

Participants passively viewed shapes presented on the computer screen and did not need to make any responses. Three shapes (+, o, and = characters) were randomly allocated to serve as one of the three different prediction cues. The M1 cue was followed by stimulation of the M1 coil 100% of the time, the P4 cue was followed by stimulation of the P4 coil 80% of the time and M1 coil 20% of the time, and the control cue was followed by nothing 80% of the time and stimulation of the M1 coil 20% of the time. The assignment of shapes to cues was counterbalanced between participants. Each cue was presented for 1200 ms, and TMS was triggered after 800 ms. Presentations of the cues were pseudorandomised such that no single cue could appear more than five times in a row. Cues were separated by a variable intertrial interval (ITI) between 2000 and 3000 ms. Baseline TMS trials (unpredictable condition) occurred between two ITI periods with no change to the screen presentation such that the time between pulses for all conditions (M1, P4, control, baseline) was all equivalent. Note that the average time between TMS was slightly longer than the average trial length since stimulation did not occur on every trial (i.e., 80% of control cues). The experiment was split into 10 blocks of 60 trials (20 of each condition) and participants had the option to continue or take a break at the end of each block. If participants requested a break, the TMS coil was taken off, so they could stretch and move their heads. The first block started with a buffer sequence that contained 12 additional trials to establish the cuing relationship and ensure the infrequent outcome did not occur (4 M1 cues that triggered M1, 4 P4 cues that triggered P4, and 4 control cues that did not trigger TMS were presented in a random order). At the beginning of the experiment, participants were instructed to pay careful attention to the shapes that appeared because they would be asked some questions at the end of the experiment. The questions acted as an attention check since there were no response requirements to assess non-compliance or if participants stopped attending during the experiment. If participants were unable to identify the relationship between the shapes and the location of stimulation, they were excluded from the data analysis. Piloting suggested that this relationship should be easily detected if participants were attentive during the task. Four participants could not identify the relationship of any of the three shapes, and their data were not included in the analysis. The total session was approximately 1.5 hrs including briefing, TMS setup, and debrief.

#### 2.7.2 Experiment 2

Two shapes (+ and o presented as in Experiment 1) were randomly allocated to serve as cues to signal the two different prediction conditions: the self-generated cue remained on the screen until participants pressed the space key with their left thumb (a different hand and finger to where MEPs are being recorded: right FDI), and TMS was triggered immediately (i.e. within milliseconds) for 50% of these key presses (such that there were equal numbers of self-generated TMS and self-generated no-TMS trials); the warning cue remained on the screen for 1500 ms, and TMS was triggered on 50% of the warning cues (such that there were equal numbers of warning cue TMS and warning cue no-TMS trials). On trials where TMS was delivered during the warning cue, stimulation was triggered after a duration equal to the median reaction time sampled from the self-generated condition; if no reaction time had yet been sampled, the delay was set to 500 ms until a response was recorded. Presentation of the cues was pseudorandomised such that no single cue could appear more than four times in a row. Cues were separated by a variable ITI between 2000 and 3000 ms. Baseline trials occurred between two ITI periods with no change to the screen presentation; TMS was triggered on 50% of the baseline trials (unpredictable condition) and the remaining 50% of trials were used as no-TMS control trials. Although TMS occurs with a probability of 0.5, this is not “chance” per se because TMS pulses are much less frequent in the absence of a visual cue or action. That is, the action and the external cue are still highly informative in signalling an increase in the probability of TMS relative to the overall base-rate probability throughout the experiment. The experiment was split into 9 blocks of 36 trials (12 of each condition) and participants had the option to continue or take a break at the end of each block. The total session was approximately 2 hrs including briefing, EEG and TMS setup, cleanup, and debrief.

### 2.8 Pre-processing and analysis

#### 2.8.1 MEP

MEP pre-processing was performed using custom software written in Python (github.com/nicolasmcnair/MEPAnalysis). Trials were excluded if EMG activity pre-stimulation window from -100 to -5 ms had either an amplitude that exceeded 50 µV or a root mean square power greater than 3 standard deviations above the mean of all trials for that participant. Mean MEP amplitudes by condition were calculated from the remaining trials using peak-to-peak difference values. Following our earlier work (Tran et al., 2019, 2020), we normalised the target condition to the baseline condition—calculating a ratio of the mean predictable MEP amplitude to mean unpredictable baseline MEP amplitude, so that attenuation was expressed proportional to the participant’s corticospinal excitability—then took the log of this ratio to correct for positive skew (see Tran et al., 2021 for further explanation). Log-normalised values significantly below 0 indicate an attenuation effect compared with baseline. Log-normalised values significantly different from each other indicate a condition effect. We have simulated how unequal trial numbers may affect log-normalisation (Livesey et al., 2025). In Experiment 1, there are more M1 cue trials than baseline trials and log-normalised MEP may tend towards being more positive. If anything, this bias may lead to an underestimate of attenuation in the M1 condition. Thus, if this condition exhibits the strongest attenuation, we can be very confident that these differences are genuine and not an artefact of data processing. Since the critical tests in Experiment 1 rely on pairwise comparisons (P4 vs. M1; P4 vs. control), we conducted planned t-statistics with a Bonferroni correction applied to the *p*-values, where *p_bonf_* < 0.05 indicates significance after adjusting for comparing a family of three comparisons (M1 vs. control; M1 vs. P4; P4 vs. control).

#### 2.8.2 EEG

For Experiment 2, EEG pre-processing was performed using EEGLAB toolbox (v2022.0; Delorme & Makeig, 2004) in MATLAB (R2022a, MathWorks, Natick, MA). Data encompassing TMS pulse artefacts were first replaced using temporal interpolation via ARfit Studio (Schneider & Neumaier, 2001). A learning window from -100 to -5 ms of the TMS pulse was used to inform an autoregressive model. This was then used to replace the data in a window starting at -5 ms up until 45 ms (determined on an individual participant basis). Finally, the original data were blended with the model over an additional period of 5 to 10 ms for all participants. The blended data comprised a linear mix over the selected time period comprising the predicted signal [83% 67% 50% 33% 17%] and original signal [17% 33% 50% 67% 83%]. The overall interpolation window was selected on a participant basis to be as short as possible. The primary event of interest was the N1 component of the AEP, where the strength of sensory attenuation has been linked to schizophrenia (Ford et al., 2007; Oestreich et al., 2015). Studies have also found sensory attenuation reflected in the P2 component in healthy adults, but its magnitude is not predictive of schizophrenia. Therefore, we analysed P2 as a secondary event of interest. Both these components were unaffected by the interpolation window that removed the TMS pulse artefact. The purpose of removing the artefact was for visualisation purposes to scale the N1 and P2 components within an appropriate range. The data were then bandpass filtered (0.1–30 Hz cutoff frequencies) and downsampled to 250 Hz. Line noise (50 Hz) was removed using the Cleanline function from the PREP Pipeline plugin (Bigdely-Shamlo et al., 2015). Bad channels were identified using the Clean Rawdata plugin based on the presence of flatline data (>5 s), remaining line noise (>5 SDs compared with signal), or lack of correlation with nearby channels (r < 0.8; Mullen et al., 2015). These were then replaced with interpolations derived from the other electrodes, weighted by distance. Noisy sections of data were cleaned using artefact subspace reconstruction (ASR) from the Clean Rawdata plugin (Mullen et al., 2015). Data were then re-referenced to the average, before epoching 1000 ms prior to and after each event marker—baseline corrected from -1000 to 0 ms of the interpolated waveforms.

Independent components analysis (ICA) was used to identify and remove artefactual components (eye blinks, muscle activity, etc.) with the assistance of the ICLabel plugin (Pion-Tonachini et al., 2019). The resulting data were imported into ERPLAB (v9.0; Lopez-Calderon & Luck, 2014), and re-epoched from 200 ms before to 800 ms after each event marker. Finally, individual epochs were removed if they exhibited a peak-to-peak amplitude difference of larger than 100 μV in a 200 ms moving window, or a sample-to-sample difference of greater than 30 μV. Six participants were excluded for having less than half the total number of trials remaining after artefact rejection. Based on a visual inspection of the waveforms and scalp topographies, two participants were found not to generate a clear N1 component and were excluded from the analysis.

Difference waves were created by subtracting the average of the non-TMS trials from the TMS trials for each of the self-generated, warning cue, and baseline conditions. The purpose of subtracting non-TMS trials is to remove any EEG activity that is unrelated to the effect of TMS. This step is most critical for the self-generated and warning cue conditions, which involve a key press and/or the onset of a visual stimulus. We note that this subtraction logic assumes that neural inputs are additive, however, we have adhered to the same protocols used in the sensory attenuation literature. The N1 component was identified for each individual by detecting the largest negative deflection in a window from 50 to 150 ms post-stimulus onset over electrodes Fz, FCz, and Cz in their grand average ERP waveform (i.e., averaged across all conditions). The boundaries of the component were then identified by finding the two time points—earlier and later than the peak—where the amplitude reached half its maximum. For each condition, the area under the curve (AUC) was then calculated between these two time points. The P2 component was identified by detecting the largest positive deflection in a similar manner between 150 and 250 ms post-stimulus.

#### 2.8.3 MEP × ERP

To examine the relationship between motor attenuation in MEPs and sensory attenuation in ERPs, delta scores were computed from raw MEPs and AUC of the N1 and P2 components. For the self-generation condition: ΔMEP scores were calculated for each participant as the difference between the mean baseline MEP and the mean self-generation MEP; ΔN1 and ΔP2 scores were calculated for each participant as the difference between the baseline AUC and the self-generation AUC. For the warning cue condition: ΔMEP scores were the difference between the baseline and warning cue MEP values; ΔN1 and ΔP2 scores were the difference between the baseline and warning cue AUC values.

## 3 Results

### 3.1 Experiment 1

**Fig. 1.**
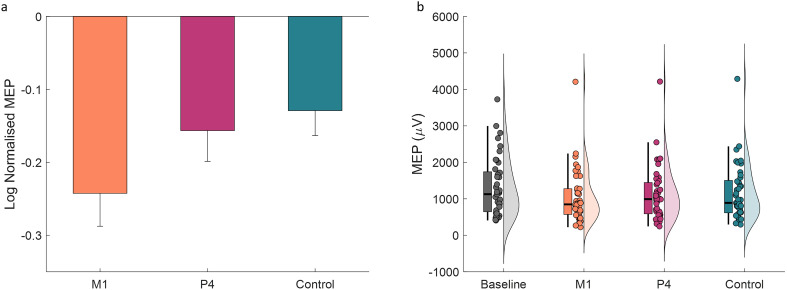
Mean log-normalised MEPs to the baseline condition (a) and raw MEPs (b). Error bars represent standard error of the means. The M1 cue was followed by motor cortex stimulation 100% of the time, while the P4 and control cues were followed by motor cortex stimulation 20% of the time. MEP = motor-evoked potential; M1 = primary motor cortex location; P4 = P4 10/20 standard EEG electrode location. Note the very positive data points (>3500 µV) in panel b are from the same participant and do not meet exclusion (>3SD) when normalising to the baseline condition for analysis.

All cues produced attenuated MEPs relative to the unpredictable baseline condition (see Table 2, left). Replicating our past probability finding (Tran et al., 2021), there was weaker attenuation for the control cue that predicted stimulation of the motor cortex 20% of the time (and nothing 80% of the time) than the M1 cue that predicted stimulation of motor cortex 100% of the time. Critically, the amount of attenuation for the P4 cue was similar to that for the control cue and weaker than attenuation for the M1 cue (see Fig. 1). This result suggests that prediction of motor cortex stimulation specifically, rather than prediction of the sensory by-products of TMS, determined the strength of attenuation. Statistically, there was a main effect of Cue Type, *F*(2,40) = 7.20, *p* = 0.001, *d* = 0.43, η^2^ = 0.15. Critically, the M1 cue showed significantly more attenuation than the control cue, *t*(40) = 3.64, *p* = 0.001, *d* = 0.57, and P4 cue, *t*(40) = 2.76, *p* = 0.022, *d* = 0.43. The P4 cue and the control cue were not significantly different, *t*(40) = 0.88, *p* = 1.00, *d* = 0.14, BF_01_ = 3.85.

**Table 2.** One sample tests of the attenuation effect compared with the baseline condition (H_0_ = 0) for Experiments 1 and 2.

	Experiment 1			Experiment 2	
	M1	P4	Control	Self	Cued
t	5.37	3.71	3.78	4.99	6.71
df	40	40	40	34	34
*p*	<0.001	<0.001	<0.001	<0.001	<0.001

*Note.* Significant values indicate that the respective condition is significantly attenuated relative to the baseline condition (i.e., log-normalised values less than 0).

### 3.2 Experiment 2

#### 3.2.1 MEP

**Fig. 2.**
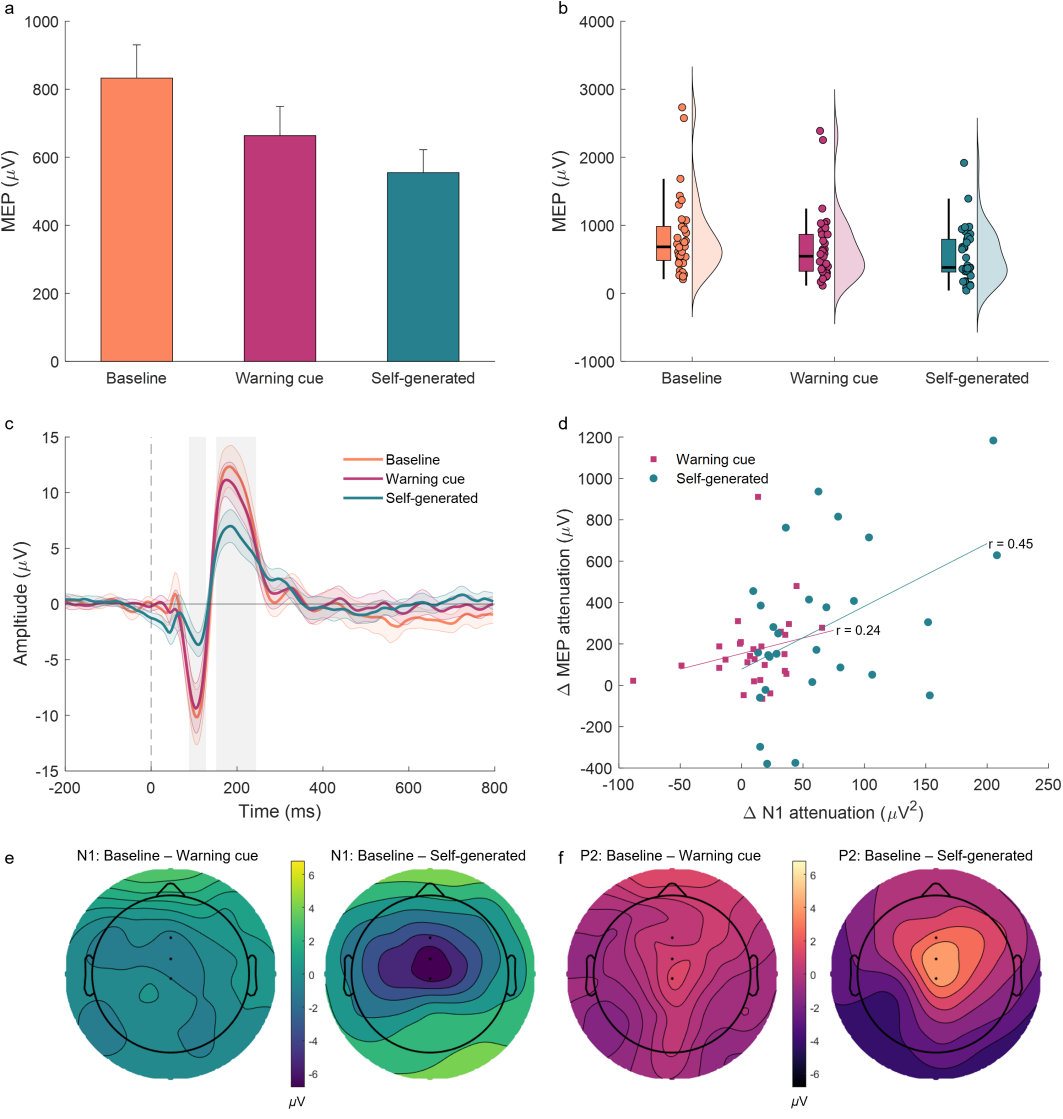
Mean and SEM of raw MEPs by prediction condition (a), and individual raw MEPs represented as raincloud plots (b). Mean and SEM of ERPs by prediction condition averaged over electrodes Fz, FCz, and Cz (c), grey bands represent the N1 and P2 time windows; and scatterplot comparing change in N1 attenuation with change in MEP attenuation (d), delta scores represent the difference between baseline and prediction conditions. Topographic difference maps of the N1 component (e) and P2 component (f) with selected Fz, FCz, and Cz electrodes. MEP = motor-evoked potential; ERP = event-related potential.

Both the self-generated and warning cue conditions produced attenuated MEPs relative to the unpredictable baseline condition (see Table 2, right). Replicating our past agency finding (Tran et al., 2021), there was stronger attenuation for the self-generated condition than the warning cue condition (see Fig. 2), *t*(34) = 2.64, *p* = 0.012, *d* = 0.45, BF_10_ = 3.58.

#### 3.2.2 ERP

Based on the peak detection method, the average latency of the N1 peak was mean = 104.52 ms, SD = 13.18 ms, and the average latency of the P2 peak was mean = 181.73 ms, SD = 24.45 ms. The self-generated and warning cue conditions were attenuated relative to baseline at both the N1 and P2 components (see Fig. 2). The differences in AUC between the baseline and self-generated conditions were significantly less than 0 at N1: *t*(30) = 6.57, *p* < 0.001, *d* = 1.18; and significantly greater than 0 at P2: *t*(30) = 7.77, *p* < 0.001, *d* = 1.40. The differences in AUC between the baseline and warning cue conditions were numerically smaller in magnitude and significantly less than 0 at N1: *t*(30) = 1.90, *p* = 0.033, *d* = 0.34; and significantly greater than 0 at P2: *t*(30) = 2.79, *p* = 0.005, *d* = 0.50.

#### 3.2.3. MEP and ERP relationship

There was a positive relationship between the magnitude of motor attenuation and sensory attenuation at the N1 component, indicating that stronger attenuation in MEP is related to stronger attenuation in N1 (larger differences equals more attenuation). The strength of attenuation of self-generated condition was significantly correlated across MEPs and the N1 component of AEPs, *r* = 0.45, *p* = 0.016, *n* = 28. The strength of attenuation for the warning cue condition was weaker and not significantly correlated across MEPs and the N1 component of AEPs, *r* = 0.24, *p* = 0.223, *n* = 28. Importantly, the differences in MEPs across conditions and the differences in AEPs across conditions were not correlated with TMS stimulation intensities (*p’*s > 0.115).

There was a negative relationship between the magnitude of motor attenuation and sensory attenuation at the P2 component, indicating that stronger attenuation in MEP is related to stronger attenuation in P2 (the negative relationship is due to the opposite sign for P2 differences, such that a more negative value for the P2 difference equals more attenuation). The strength of attenuation for the self-generated condition was negatively correlated, but not significant, across MEPs and the P2 component of AEPs, *r* = -0.28, *p* = 0.153, *n* = 28. The strength of the attenuation for the warning cue condition was also not significant across MEPs and the P2 component of AEPs, *r* = -0.18, *p* = 0.353, *n* = 28.

As a supplementary analysis, we analysed the MEP attenuation data and its relationship with N1 and P2 attenuation using linear mixed effects models, providing a way of pooling the self-generated and warning cue conditions while accounting for within-participant dependency. Predicting MEP attenuation from N1 attenuation, prediction condition, and participants (as a random effect) also returned N1 as a significant predictor, β = 3.15 µV, SE = 0.78, t(49.67) = 4.07, *p* < 0.001. No other predictors in this model were significant. Predicting MEP attenuation from P2 attenuation, prediction condition, and participants (as a random effect) did not return P2 as a predictor, β = -0.71 µV, SE = 0.50, t(46.62) = -1.41, *p* = 0.17. Other predictors in this model were also not significant. In sum, when accounting for within-participant dependency using a linear mixed effects model, N1 attenuation remained a significant predictor of MEP attenuation, but P2 attenuation did not reliably predict MEP attenuation.

## 4 Discussion

The results presented here replicate our initial demonstration that the motor system responds less to predictable stimulation of the primary motor cortex compared with unpredictable stimulation (Tran et al., 2021; Tran & Livesey 2021; see also Sriutaisuk & Franz, 2025; Takei et al., 2005). When participants self-generated TMS or were presented with a warning cue that signalled the imminent arrival of TMS, MEPs in both predictable conditions were attenuated compared with MEPs triggered by less predictable, unsignalled TMS.

In Experiment 1, we showed that motor attenuation is sensitive to the strength of the predictive relationship (i.e., the probability of stimulation) between a warning cue and TMS; the 100% probability cue (M1) resulted in greater attenuation than the 20% probability cue (control). The cues in this experiment are matched for motor behaviour output in that none require a response but differ only in their probability of simulation, replicating our past work (Tran et al., 2021). We have also demonstrated similar effects in a version of the task where participants made a response on every trial, but the probability of stimulation depended on the cue (Tran et al., 2021). These results show that it is predictability, rather than motor state, that is critical for seeing attenuation. Using two coils to trigger single-pulse TMS at distinct locations, we confirmed that the motor attenuation effect is driven by prediction of direct stimulation of the motor cortex, rather than an epiphenomenon caused by predicting the sensory by-products of TMS (control vs. P4 comparison).

The two-coil setup from Experiment 1 rules out alternative explanations for the motor attenuation effect. Our previous results (Tran et al., 2021) indicated that interhemispheric inhibition (caused by initiating TMS with a press) was unlikely to play a strong role in the prediction effect, firstly because the effect is still robust even when participants make no responses at all, but also because attenuation was the same when generating TMS with a near-homologous effector (left thumb) versus non-homologous effector (left foot). If the attenuation effect was driven by interhemispheric inhibition, we should see stronger attenuation in the near-homologous effector than the non-homologous effector. However, we acknowledge that these controls do not completely eliminate motor-state differences. Tran et al. (2021) also found that attenuation was not based on arousal because the pre-EMG activity prior to the TMS was comparable between predictable and unpredictable stimulation. Notably, the two-coil setup of Experiment 1 corroborates these explanations by using a cueing design that does not require engagement of the motor system and by matching the sensory basis for heightened arousal (i.e., anticipating the sound and feel of the TMS) across the M1 and P4 cues. Another alternative explanation is that the attenuation effect could be driven by proprioceptive feedback from the finger twitch. However, the sensation and observation of a finger twitch vary greatly between individuals, and, therefore, we think it is unlikely to drive the effect.

In Experiment 2, we found that the typical sensory attenuation effect in AEPs can be replicated using the click sound of a TMS coil that is produced when delivering stimulation. We also found that individual differences in sensory attenuation predicted the strength of motor attenuation, such that individuals who showed greater sensory attenuation at N1 and P2 tended to show greater motor attenuation measured simultaneously in MEPs. For the N1 result, which was our primary event of interest, prediction via self-generation predicted motor attenuation but prediction via external cueing did not significantly predict motor attenuation. Of course, finding the relationship across both prediction conditions would provide stronger evidence for commonalities between motor and sensory attenuation effects. However, there are differences in the strength of and inter-individual variance in attenuation resulting from self-generation versus external cueing that, for our sample size, would likely make it easier to observe a correlation for the self-generated condition. The linear mixed effects model partly addresses some of these concerns; when accounting for within-participant dependency and prediction condition, N1 attenuation remained a significant predictor of motor attenuation. The P2 attenuation result, however, did not significantly predict motor attenuation. This dissociation between N1 and P2 mirrors the findings from clinical research showing that the strength of N1 but not P2 attenuation is predictive of schizophrenia symptomatology (Ford et al., 2007; Oestreich et al., 2015).

Experiment 2 also replicated the finding that self-generated predictions produce stronger attenuation than cued predictions using a visual warning signal. This pattern of results was observed in the motor domain (as in Tran et al., 2021) and the sensory domain (as in e.g., Harrison et al., 2021). When matching for motor activity state, the strength of prediction (e.g., probability) affects the magnitude of attenuation. Therefore, we interpret the stronger attenuation from self-generated predictions over cued predictions being due to self-generated predictions providing more precise temporal information about when the stimulation will occur. Self-generated predictions trigger stimulation almost instantaneously from the time of the key press, while visually cued predictions involve some temporal judgement or time estimation, which is more error prone. However, it is also possible that some of the self-generated attenuation is due to the act of making a response, whereby motor preparation and recruitment lead to inhibition (see above for discussion on why interhemispheric inhibition is unlikely to drive the attenuation effect). These concerns are also largely mitigated in the EEG data as the AEP comprises difference waves that subtract the waveform from no-TMS conditions from the waveform of the TMS conditions. That is, the self-generated AEP represents the difference between self-generated TMS trials and self-generated no-TMS trials, subtracting any activity generated from the action of pressing. This subtraction was also applied to the warning cue and baseline conditions, yet the order in the strength of attenuation remained the same as the MEP data. For these reasons, we do not think that the difference in self-generated attenuation compared with visually cued attenuation is entirely due to the involvement of an action. Nevertheless, we note that this subtraction logic does assume that neural inputs are additive, and it is possible that the increased motor attenuation is due to a combination of more precise prediction as well as motor recruitment and preparation.

The effect of prediction and expectation on motor networks has been examined in the past, but not in the way we have done here, using TMS as both the input to the motor system and the direct source of the motor system’s output. As an input, TMS acts as the event to be predicted. As the direct cause of the output, TMS generates a measure (MEP amplitude) of the motor response to the predictable versus unpredictable event. Previous studies examining the effect of surprising or unexpected events on the motor system have measured motor activity on a time scale that permits cognitive systems responsible for attention orienting and inhibitory control to process that event before MEPs are measured. For example, presenting a surprising visual or auditory event and following that up with TMS 150–200 ms later (Wessel & Aron, 2013). Over this time, it has been reported that unexpected events result in a global suppression of the motor network (Wessel & Aron, 2017). This, of course, is the opposite pattern to what we find when the TMS *itself* is unexpected, as in this case the unexpected event results in a larger MEP. A key difference in the protocols is that TMS provides a measure at the time of stimulation; it is not simply a tool to measure the downstream consequences of a surprising or expected event but rather it *is* that event (whether it is expected or not). This has entirely different consequences for the processes that occur within the motor system in response to TMS, and it may well be the case that an unexpected pulse of TMS triggers a large MEP *before* attention orienting and other cognitive processes lead to a general suppression of the motor system. It should be noted that the motor suppression effect shortly following unexpected events is partly driven by the event being infrequent, rather than the event being unpredictable (Iacullo et al., 2020). In contrast, our TMS predictability effects are present even when controlling for the (in)frequency of the event (Sriutaisuk & Franz, 2025; Tran et al., 2021).

The current findings demonstrate the reliability and robustness of prediction-based motor attenuation measured using TMS. If the results produced with TMS represent how the motor system responds to patterns of neural activity more generally (e.g., motor signals, or internally generated neural noise), then it may indicate that the motor system attenuates firing to more predictable signals relative to less predictable signals. This raises the question: Why might the motor system operate in this way? The functional relevance of down-weighting predictable motor activity is not immediately obvious. Attenuating predictable sensory activity has clear functional and adaptive benefits as it helps organisms differentiate novel and unexpected information from redundant and repetitive information (Crapse & Sommer, 2008); it also helps organisms suppress expected tactile or visual feedback from one’s own actions via an afferent system (Blakemore et al., 1999). We have previously hypothesised that prediction-based motor attenuation may function to conserve metabolic energy and enhance detection of signal from noise (Tran et al., 2021; Tran & Livesey, 2021). Recent work suggests that task-irrelevant motor movements can overwhelm neural activity (Mathis, 2019; Musall et al., 2019) and these results give credence to the signal detection idea we previously proposed. If organisms can anticipate upcoming motor signals, attenuating these predictable signals can free up the brain to process novel, relevant information. For example, if repetitive motor ticks or habitual actions can be attenuated, any abrupt or spontaneous motor commands can be executed in a more controlled and precise manner. Alternatively, it is possible that attenuating predictable motor signals does not serve a separate adaptive function, but is instead a result of exaptation from existing neural mechanisms controlling functions in other neural systems (e.g., attenuation in the sensory domain). An exciting corollary of this idea is that down-weighting predictable activity is a domain-general property of neurons, not specific to the sensory or motor systems.

A number of highly influential frameworks have described sensory processing, particularly visual perception, in terms of predictive coding (Friston, 2005; Rao & Ballard, 1999). The common theme in these frameworks is that the brain is constantly generating controlled, top-down predictions to construct mental models about the external world that are compared with incoming, bottom-up information. Mismatches between these top-down predictions and bottom-up sensory information give rise to prediction errors that signal the mental models require updating. Neural attenuation of predictable information may, therefore, be a way to increase coding efficiency and maximise sensitivity for detecting prediction errors. Our current results suggest that predictions based on a mental model and comparisons with incoming information need not be confined to sensory processing, but a similar mechanism can be observed when processing motor information. Supporting this thesis, we found that the magnitude of sensory attenuation at the N1 component predicted the magnitude of motor attenuation in MEPs. While a correlation between measures does not imply a shared causal mechanism, we have ruled out a number of alternative hypotheses and found that the variance is not related to cortical excitability as determined by resting motor thresholds. However, we note that other plausible accounts for observing a relationship between sensory and motor attenuation include covariations in attention or proactive inhibition. Being able to systematically rule out these possible explanations would strengthen the interpretation that there are variations among healthy individuals in the degree to which prediction modulates neural responses irrespective of the domain in which that information is processed.

Individual differences in neural responsiveness to predictive information has been shown to influence psychological and cognitive function. For example, individuals with schizophrenia do not display the typical N1 suppression effect to predictable sounds (Ford et al., 2007), and schizotypy traits positively correlate with weaker N1 suppression (Oestreich et al., 2015). These impairments in sensory attenuation among individuals with schizophrenia have been hypothesised to account for the presence of delusions of control and even auditory hallucinations, whereby self-generated (predictable) actions or sounds are not sufficiently attenuated and have neural representations that are much more similar to those of externally generated events. If the capacity to use predictive information to down-weight anticipated neural activity varies meaningfully across individuals, and the inability to do so can explain schizophrenia symptomology, deficits in processing predictable information across domains may underlie other clinical disorders. Indeed, impairments in reward prediction error have been reported in individuals with major depressive disorder and bipolar disorder (Whitton et al., 2015). Finding evidence for predictive coding as a domain-general principle has important implications for understanding the origins of transdiagnostic and co-occurring symptoms in populations with mental health conditions. Previous researchers have also argued for a general prediction deficit over a domain-specific deficit as a basis for hallucinations and psychosis (Corlett et al., 2019; Powers et al., 2017; Sterzer et al., 2018). However, to our knowledge, no published work has directly tested these ideas by examining how predictions across different systems relate to each other.

Using two-coil TMS and combined TMS-EEG we were able to isolate the motor attenuation effects to direct stimulation and link attenuation effects across sensory and motor domains. However, both techniques are relatively novel, so it is worth discussing whether the protocols are working as intended, particularly with TMS-EEG. Combining TMS-EEG allowed us to measure AEPs to the sound accompanying TMS stimulation. However, combined TMS-EEG is often used for measuring TMS-evoked potentials (TEPs)—cascades of neural activity resulting from stimulating the brain (Ilmoniemi & Kičić, 2010). For our purposes, it is important to establish, firstly, that the TMS coil click can generate a clean auditory-evoked potential with distinct N1 and P2 components; and secondly, that the N1 and P2 components are not solely the product of TEPs. Both these considerations were tested by Rocchi et al. (2021) who used noise masking to manipulate the effect of the coil sound on the EEG recording. The authors showed that the sound of the TMS coil can elicit a clean auditory-evoked potential with a negative peak around 100 ms (N1 component) and a positive peak around 200 ms (P2 component). They also found that early activity between 15 and 65 ms was largely due to TEPs, while middle activity between 65 and 120 ms around N1 was largely due to AEPs, and late activity between 120 and 270 ms around P2 was entirely due to AEPs. Therefore, we can be confident that our N1 and P2 components reflect neural activity in response to the sound of the coil click rather than a neural activity from brain stimulation.

In summary, our results show that prediction in the motor system can be measured with TMS. The results provide further evidence that prediction-based attenuation can be observed across different neural systems, in sensory, reward, and now motor networks. Most interestingly, we showed that the strength of neural attenuation in one domain predicted attenuation in another, with prediction effects in AEPs related to prediction effects in MEPs. These findings provide promising evidence consistent with our hypothesis that attenuating neural activity to predictable or expected information may be a domain-general property of the brain and provide novel insights for how predictive coding mechanisms may be affected in clinical disorders.

## Data Availability

The data are available at https://osf.io/uv75c
